# Diversification and reproductive isolation: cryptic species in the only New World high-duty cycle bat, *Pteronotus parnellii*

**DOI:** 10.1186/1471-2148-13-26

**Published:** 2013-01-29

**Authors:** Elizabeth L Clare, Amanda M Adams, Aline Z Maya-Simões, Judith L Eger, Paul DN Hebert, M Brock Fenton

**Affiliations:** 1 Department of Integrative Biology, University of Guelph, Guelph, Ontario, Canada; 2 School of Biological Sciences, University of Bristol, Bristol, UK; 3 Department of Biology, Western University, London, Ontario, Canada; 4 Department of Natural History, Royal Ontario Museum, Toronto, Ontario, Canada; 5 Universidade do Estado do Rio de Janeiro, Rio de Janeiro, Rio de Janeiro, Brazil

**Keywords:** Cryptic species, DNA barcoding, Systematics, Bats, Biodiversity, Speciation, *Pteronotus mesoamericanus*

## Abstract

**Background:**

Molecular techniques are increasingly employed to recognize the presence of cryptic species, even among commonly observed taxa. Previous studies have demonstrated that bats using high-duty cycle echolocation may be more likely to speciate quickly. *Pteronotus parnellii* is a widespread Neotropical bat and the only New World species to use high-duty cycle echolocation, a trait otherwise restricted to Old World taxa. Here we analyze morphological and acoustic variation and genetic divergence at the mitochondrial COI gene, the 7^th^ intron region of the y-linked *Dby* gene and the nuclear recombination-activating gene 2, and provide extensive evidence that *P. parnellii* is actually a cryptic species complex.

**Results:**

Central American populations form a single species while three additional species exist in northern South America: one in Venezuela, Trinidad and western Guyana and two occupying sympatric ranges in Guyana and Suriname. Reproductive isolation appears nearly complete (only one potential hybrid individual found). The complex likely arose within the last ~6 million years with all taxa diverging quickly within the last ~1-2 million years, following a pattern consistent with the geological history of Central and northern South America. Significant variation in cranial measures and forearm length exists between three of the four groups, although no individual morphological character can discriminate these in the field. Acoustic analysis reveals small differences (5–10 kHz) in echolocation calls between allopatric cryptic taxa that are unlikely to provide access to different prey resources but are consistent with divergence by drift in allopatric species or through selection for social recognition.

**Conclusions:**

This unique approach, considering morphological, acoustic and multi-locus genetic information inherited maternally, paternally and bi-parentally, provides strong support to conclusions about the cessation of gene flow and degree of reproductive isolation of these cryptic species.

## Background

### Mammal species diversity in the Neotropics

Central and northern South America are frequently cited as hotspots for speciation due to complex biological and geological processes in this area. The rise of the Central American land bridge some 3 million years ago caused a sudden mixing of previously isolated terrestrial species in the Great American Interchange while simultaneously isolating marine populations on the Atlantic and Pacific sides [1]. The rise of the Andes Mountains, including the Eastern Cordillera ending some 2–3 million years ago [2], served to isolate previously contiguous terrestrial populations, particularly those with low dispersal abilities. Given these two geological events, it is not surprising that this geographical area has been the focus of many phylogeographic and taxonomic investigations in a wide variety of taxa. Species with large body size tend to have greater dispersal ability [3] and volant species may be particularly capable of overcoming dispersal barriers [4], likely intermixing populations from Central and South America more easily. Theoretically, increased dispersal ability may result in more homogenized populations [5] reducing population structure and thus speciation. Despite this, the only volant mammals, bats, are frequently found to have significant population divisions, subspecies, and sister-species relationships between Central and South American taxa [6-9].

Avise and Walker [10] hypothesized that half of all vertebrates remain undescribed. This level of cryptic diversity might be expected among groups that have received little taxonomic scrutiny, such as invertebrates (e.g. [11,12]), but the presence of new species among large, charismatic, well-studied taxa is less expected. Despite this, an average of 223 mammalian species have been described each decade since the beginning of Linnaean taxonomy and the rate of discovery has increased recently, particularly in areas of high endemism [13]. The contemporary discovery of new species rarely reflects the discovery of novel morphological forms or the rediscovery of species thought extinct (though see [14]). Instead, new species are generally uncovered during reassessments of existing taxa using multiple lines of new evidence (behaviour, ecology, molecular markers), in conjunction with traditional morphological analysis. While morphological differences may be subtle and thus easily overlooked, high genetic divergences within species often indicate cryptic diversity and can direct taxonomic analysis. Evidence of undescribed species has been found even among well-studied mammals, such as orangutans [15], warthogs [14], giraffes [16], muntjacs [17], baleen whales [18], beaked whales [19] and elephants [20] and further cases are to be expected among less known taxa. Among mammals, bats are the second largest order with more than 1100 described species [13]. They are an obvious target for the discovery of new species as their cryptic behaviour (e.g. nocturnal and active high in the air) makes them difficult to study in the wild [21]. Genetic analysis has detected cryptic species among the European bat genus *Pipistrellus*[22], the widely distributed genera *Plecotus* and *Myotis*[23] and overlooked species appear common in both Southeast Asia [24] and the Neotropics [7-9,25,26].

### Rapid speciation in bats with high-duty cycle echolocation

The majority of bats use acoustics, mainly echolocation, as a primary means of orientation, prey detection, and social recognition. Two forms of echolocation exist among bats: low-duty cycle echolocation entails separating pulse and echo in time, while high-duty cycle echolocation employs Doppler shift compensation to separate pulse and echo in frequency. High-duty cycle echolocators have an “acoustic fovea” which allows them to resolve fine differences in frequency [27-29]. High-duty cycle echolocation occurs in ~120 species in the Old World families Rhinolophidae and Hipposideridae [30]. Only one species in the New World, *Pteronotus parnellii* (family Mormoopidae), has evolved high-duty cycle echolocation with the most energy of the call in the second harmonic, ~61.5 kHz [31], with harmonics at ~30 kHz intervals.

Among echolocating bats, acoustic traits may diverge by drift in allopatric populations (see discussion in [32]); however, it has been argued that selection for non-interference between inter-population calls in sympatric zones may also drive speciation [33] through local adaptation [32]. Four hypotheses for speciation and echolocation have been proposed. First, call divergence can occur through disruptive ecological selection on frequency leading to prey specialization and gradually to reproductive isolation [33] with speciation as a by-product of acoustic resource partitioning. High frequencies are more effective at detecting small insects and orienting in cluttered environments while low frequency calls provide more details on large insects and over longer distances, though reasonably large differences in emission frequency are required for functional differences in target identification [34,35] due to the relationship between wavelength and target detection of insect sizes (see [29] for complete details). As a result of this selective pressure we expect large acoustic variation between competing species in order for them to access different sized prey (e.g. 10 kHz difference in frequency translates into only a small difference in target strength detection; see [33,35]). Second, selection for non-interference in acoustic signals between populations may cause similar patterns of acoustic divergence. In two morphs of *Hipposideros bicolor,* Kingston *et al.*[33] noted substantial acoustic divergence but not enough (<10 kHz) for significant resource partitioning. Kingston *et al.*[33] suggest that selection has acted against signal interference yielding character displacement due to social interactions. Under this hypothesis, we would predict small but consistent variation in echolocation frequency (<10 kHz). Third, acoustic divergence can cause both resource and social isolation simultaneously if populations specialize on a lesser-used harmonic of their fundamental. For example, species in the high-duty cycle *Rhinolophus philippinensis* complex use alternate harmonics consistent with a hypothesized switch via “harmonic hopping” creating an almost instantaneous method of reproductive isolation as the taxa become unable to detect each others’ calls [29]. At the same time, switching harmonics significantly changes the insect prey detection parameters to an almost non-overlapping resource use or “ecological discontinuity” [29]. In the *Rhinolophus* case, divergence between acoustic morphs may lead to both assortative mating and selection for divergent resource preferences. Most interestingly, Kingston and Rossiter [29] note that all known echolocation in the clade occurs at a harmonic of the fundamental frequency of the large *R. philippinensis* morph suggesting rapid radiation via this mechanism. Under this hypothesis, acoustic divergence would be large (>10 kHz) but in specific harmonic intervals of the fundamental frequency of the ancestral population. Finally, allopatric populations may experience acoustic variation due to drift which may be reinforced during secondary contact. Similar to selection for non-interference, these divergences are expected to be small.

While these systems may act in low-duty cycle echolocation, Kingston *et al.*[33] argued that divergence in acoustic characters is more likely to cause fast reproductive isolation in high-duty cycle echolocation where selection for mate recognition within phonic groups is reinforced by postnatal tuning of the auditory fovea forming a positive feedback loop. Divergence in high-duty cycle echolocation may be selectively stronger since tuning of the constant frequency component and the auditory fovea for insect flutter detection constrains the use of acoustics for social purposes.

### Molecular Diversity in *Pteronotus parnellii*

Genetic analysis has helped reveal many bat species complexes (e.g. [7,9,23,33,36,37]). *Pteronotus parnellii* was described from Jamaica [31] but is widely distributed in Mexico, Central America, the Antilles, the Guyana Shield and the Amazon [31]. Molecular analysis has revealed substantial sequence divergence at mitochondrial loci [8,25,38,39] though, to date, no multi-gene analysis of this variation has been conducted.

In this study we examine specimens of *P. parnellii* from ten countries in Central and northern South America to assess morphological, genetic, and acoustic divergences within this species. Following Clare [9], we test whether *P. parnellii* is actually a complex of undescribed species by comparing patterns of divergence in maternally inherited DNA and the paternally inherited 7^th^ intron of the *Dby* region (*Ddx3y*, DEAD box RNA helicase Y) on the y-chromosome [40]. In addition, we acquired exonic sequences from the 5’ half of the nuclear recombination-activating gene 2 (RAG2) to support phylogenetic reconstructions. We predict that 1) patterns of mitochondrial divergence will be supported by divergence at non-mitochondrial loci, 2) genetic divergence corresponds to subtle morphological differences between these unrecognized species and, 3) acoustic divergences have led to identifiable phonic groups.

Given the geological barriers (such as the Andes) and isolated populations in the Antilles, we anticipate that cryptic species may exist with both allopatric and sympatric distributions and their origin may also have occurred under either of these scenarios. As such, we compare the acoustic patterns of individuals from this range and look for patterns corresponding to the competing hypotheses for acoustic divergence in allopatry and sympatry: 1) if acoustic divergence is primarily for resource partitioning in sympatry, then signal divergence between phonic groups will be large (>10 kHz), 2) if acoustic divergence has occurred through drift in allopatry or selection for non-interference between phonic groups without causing changes in resource use, variation will be significant but small (<10 kHz), and 3) if divergence follows the “harmonic hopping” model of sympatric divergence, then constant frequency components between phonic groups will occur with the most energy at a harmonic of the frequency of ~61.5 kHz attributed to this species.

## Methods

### Acquisition of samples

We sampled tissue from 343 vouchered specimens of *P. parnellii* at the Royal Ontario Museum (ROM) originating from Mexico, Guatemala, El Salvador, Panama, Venezuela, Guyana and Suriname. We analyzed an additional 106 wing biopsies from un-vouchered individuals caught in Costa Rica and Belize using harp traps and mist nets and from a group of captive individuals originating in Trinidad. Descriptions of all specimens (sampling location, GPS coordinates of collection, voucher number, etc.) are available within the “Bats of the Neotropics” project in the Barcode of Life Data Systems (BOLD, http://www.barcodinglife.org). Records for un-vouchered specimens are contained on BOLD within the projects “Bats of Belize,” “Bats of Costa Rica,” and “*Pteronotus parnellii* from the Islands.” GenBank and BOLD accession numbers for all sequences, along with sequence alignments, are found in the Additional files 1, 2, 3, 4. All live animals used during the course of this study were caught and handled following the guidelines of the Canadian Council on Animal Care (CCAC – species-specific recommendations on bats) under approval from the Animal Care Committee, University of Guelph (#08R132) and MINAET-ACG permit (FOI-00-004) (Costa Rica). All other tissues and acoustic recordings were acquired from existing collections.

### Acquisition and analysis of sequences

We used standard protocols for DNA extraction, amplification, and sequencing for a 657 bp segment near the 5′-terminus of the mitochondrial COI gene [25] for all individuals. We generated sequences from the *Dby* 7^th^ intron region of the y-chromosome from DNA extracts of 80 male specimens using the primers in Lim *et al.*[40]. For 70 male individuals we recovered 767 bp from the 5’ end of the nuclear exonic region of the RAG2 gene (region F1B to R1) using the primers and regions described by Baker *et al.*[41]. All PCR and sequencing primers are given in Additional file 5 and references therein. We edited COI sequences using SeqScape v.2.1.1 (Applied Biosystems) and *Dby* 7^th^ intron and the RAG2 region sequences in Sequencher v.4.5 (Gene Codes), and manually aligned all sequences in BioEdit v.7.0.9 (Ibis BioSciences).

### Phylogenetic reconstructions

We constructed a 95% confidence limit haplotype network of all COI sequences using statistical parsimony in TCS v.1.13 [42]. Following Clare [9], in all further analyses we define putative cryptic species or “groups” based on the presence of independent (unconnected) COI networks at the 95% confidence level.

We selected appropriate models of sequence evolution in MODELTEST [43] executed in Phylemon v.2.0 [44] using the Akaike information criterion (AIC) for each set of sequences (COI, *Dby* 7^th^ intron and RAG2 region). We reduced all datasets to unique haplotypes for phylogenetic reconstruction. For COI sequences and a combined COI + *Dby* 7^th^ intron + RAG2 sequence dataset we constructed a maximum likelihood phylogeny using PhyML v.3.0 [45] as implemented by the ATGC Montpellier Bioinformatics Platform (http://www.atgc-montpellier.fr/phyml/) using the best fit model of sequence evolution. Branch support was calculated using the non-parametric Shimodaira-Hasegawa-like (SH-like) approximate likelihood ratio test (aLRT) [46]. For the same datasets we constructed a Bayesian phylogeny partitioned by gene and codon position using alternate models of sequence evolution in MrBayes v.3.1.2 [47]. The analyses were performed for 1,000,000 generations for every 10 specimens in the analysis (e.g. for 70 specimens + 3 outgroups in the concatenated tree, runs ran for 7.3 million generations to reach stationarity) and sampled every 50 generations with a burnin of 10,000 generations using multiple outgroups (*Noctilio albiventri*s and *Saccopteryx bilineata* from South America and *Rousettus aegyptiacus* from the Old World)*.* Convergence and stationarity were compared between multiple runs. We also evaluated species trees against this concatenated gene tree with the same parameters using the *BEAST option in BEAST v.1.7.4 [48] including input files generated in BEAUTi v.1.7.4 [48].

### Estimates of divergence time

We estimated divergence times among genetic groups with RAG2 and COI sequences using Bayesian MCMC, executed in BEAST (as above). Since no estimates of COI mutation rate are available for bats, we did not use a fixed substitution rate but estimated minimum and maximum divergence times using two substitution models, 2% and 5% per million years, based on estimates for cyt *b* in small-bodied mammals [49]*.* Similar estimates of 2.6% for phyllostomid bats [6], 2.3–5% in *Carollia*[50], and 4% from fossil calibrations [51] have been suggested. We used a fixed clock of 0.194% per million years for the RAG2 sequences [52]. The nucleotide substitution model was the same as that used for phylogenetic analysis. Branching structure of the groups was fixed based on the topology supported by the multi-gene reconstruction. The chain ran for 10,000,000 generations using the 28 unique haplotypes + outgroups with a burnin of 10,000. We estimated the mean and 95% confidence interval (CI) of the divergence times using Tracer v.1.4.1 [48] and summarized the trees using TreeAnnotator v.1.4.8 [48]. We visualized the trees with FigTree v.1.2.1 (A. Rambaut 2009 http://tree.bio.ed.ac.uk/software/figtree).

### Analysis of morphology

**Figure 1:**
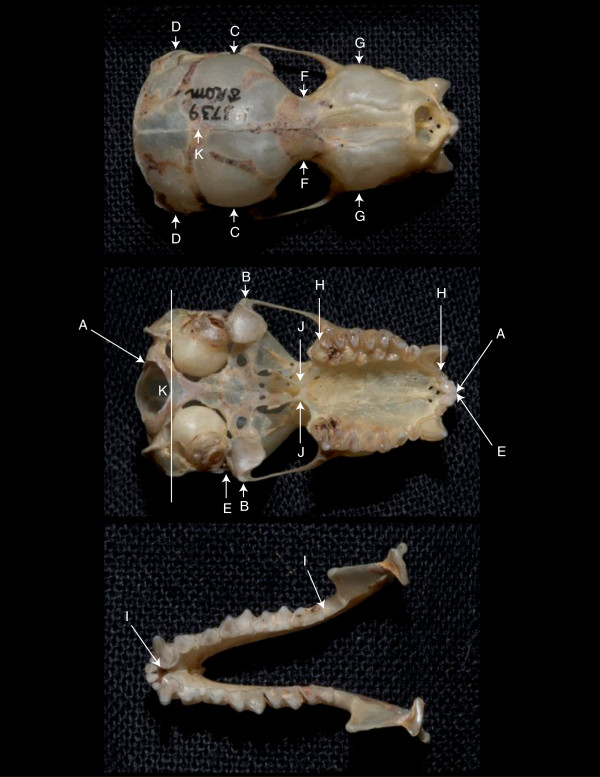
**Eleven cranial measures used in morphometric analysis of *Pteronotus parnellii *following Smith [**[53]**].** The characters are: condylobasal length (**A-A**), zygomatic breadth (**B-B**), breadth of braincase (**C-C**), mastoid breadth (**D-D**), zygorostral length (**E-E**), interorbital breadth (**F-F**), rostral breadth (**G-G**), alveolar length of maxillary toothrow (**H-H**), alveolar length of mandibular toothrow (**I-I**), breadth of post-palatal extension (**J-J**), depth of braincase (**K-K**).

We measured 11 cranial characters (Figure 1) from 165 adult, skeletal specimens from the vouchers used for genetic analysis. The measurements were made using an IP67 Special ABS Coolant-Proof Mitutoyo Caliper with a resolution of 0.01mm. All measurements were based on those described in Smith’s [53] revision of the Mormoopidae family. The “depth of braincase” measure was adapted to prevent injury to the stylohyal bone. Rather than placing the blade of the caliper across the glenoid fossae, the blade rested between the bullae and the exoccipital condyles (Figure 1). Condylobasal length and maxillary toothrow were taken parallel to the axis of the skull. We acquired forearm length for each specimen from field records. 

### Analysis of acoustic variation

We acquired echolocation calls recorded during passive monitoring using Avisoft-Bioacoustic CMPA/CM16 condenser ultrasound microphones (Avisoft Bioacoustic 2006) at sites in Jamaica (the type locality of this species), Belize, Costa Rica, southern Guyana (B Lim, pers. comm.), and Trinidad (H Goerlitz pers. comm.). The acoustic records were collected at the same location as the site of genetic sampling but cannot be traced to specific specimens. We analyzed calls using callViewer18 [54], recording the constant frequency component of each call. We analyzed all calls in a pass (>5 calls), with each pass being considered a separate individual.

### Statistical analysis

We compared forearm length among the genetic groupings with a Kruskal-Wallis test in R 2.13.1 and conducted a pair-wise post-hoc test using kruskalmc (pgirmess package) [55]. All remaining statistical analysis was done using PASW 18 [56]. Because cranial measures are internally correlated, we performed a principal components analysis (PCA) with ten of the skull measurements. We excluded the measure of post-palatal extension from analysis as it is nearly invariant between individuals from all groups. We then used discriminant function analyses (DFA) with the principal component (PC) scores generated from the PCA to classify individuals and an ANCOVA to examine the relationship between sex, latitude and PC scores. Since the groups are distributed with rough association to latitude, we regressed the PC scores against latitude and used the residuals in a DFA to see if there is still a difference among groups above the latitudinal cline.

We compared the constant frequency of the second harmonic among groups with an ANOVA and Tukey’s post-hoc test. Only the constant frequency of the second harmonic was analyzed because of the high correlation between harmonics. For all locations, the second harmonic had the highest intensity and is thus most consistently present and quantifiable. We used a DFA to see how well bats could be classified to their genetic grouping and to the five acoustically sampled locations based on the constant frequency component of their echolocation.

## Results

### Models of sequence evolution and phylogenetic reconstruction

**Figure 2:**
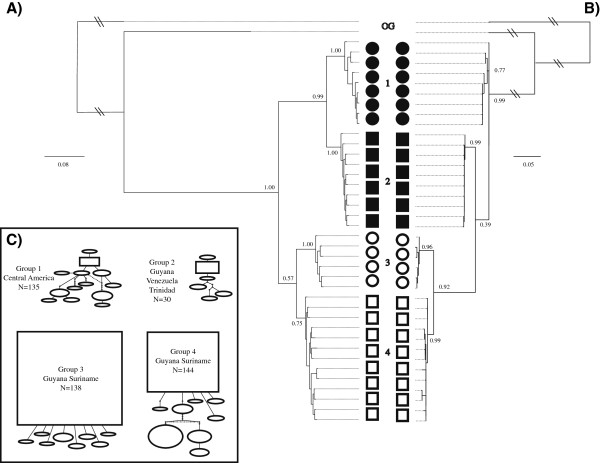
**Bayesian (A) and maximum likelihood (B) phylogenetic reconstruction of unique haplotypes of the 5’ region of COI mitochondrial lineages for the bat species *Pteronotus parnellii *.** Branch supports represent posterior probabilities and non-parametric Shimodaira-Hasegawa-like (SH-like) values respectively. Both analyses were performed on a reduced (unique haplotypes only) dataset. Four haplotype networks (**C**) correspond to the four major lineages in the phylogenetic constructions. Each circle in the network represents a single haplotype with circle size scaled by haplotype frequency. Squares indicate the most common haplotype in the network.

**Figure 3:**
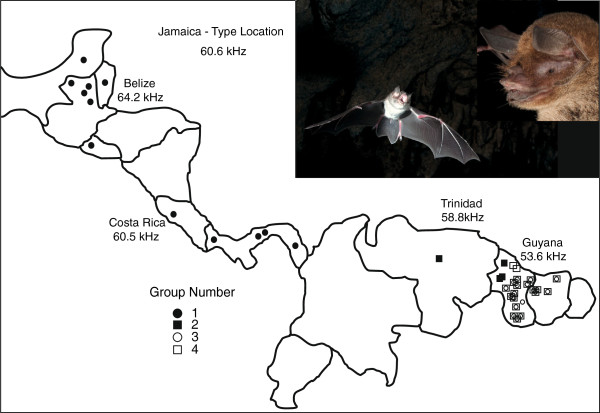
**Distribution of sampling sites for genetic groups.** Group 1 is restricted to Central America. Groups 3 and 4 occupy sympatric distributions in the lowlands of the Guyana Shield while the few individuals in Group 2 occurred at higher elevations in Guyana and in Venezuela and Trinidad. Measures of the constant frequency component of most energy for echolocation calls are indicated. Photographs by E.L. Clare and M.B. Fenton.

**Figure 4:**
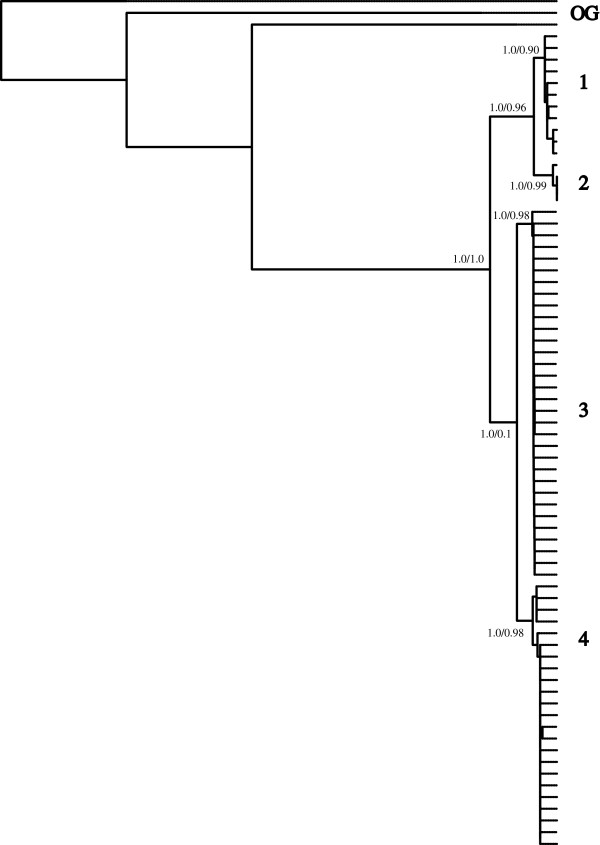
**Phylogenetic reconstruction of the combined COI, *Dby *7**^**th **^**intron and RAG2 regions supports a single topology for the bat species*Pteronotus parnellii.*** Branch supports represent posterior probabilities followed by non-parametric Shimodaira-Hasegawa-like (SH-like) values respectively.

We recovered four distinct unconnected haplotype networks in the COI sequence data (Figure 2). The AIC in MODELTEST [43] indicated an HKY+I [57] model of sequence evolution for the mitochondrial COI region, an HKY [57] model for the RAG2 region, and a GTR [58] model of sequence evolution for the *Dby* 7^th^ intron region of the y-chromosome. All phylogenetic reconstructions strongly support the existence of the four groups recognized as discrete networks (henceforth Groups 1, 2, 3 and 4), though the arrangements of these groups relative to each other differ slightly between reconstruction methods when only COI sequences are used (Figure 2). Phylogenetic analyses partitioned by codon and gene and those based on species trees from multilocus data in *BEAST [48] produced identical topologies and very similar branch support values. We conducted multiple runs for each parameter set to ensure convergence between runs. We evaluated stationarity, convergence, and burnin in MrBayes [47] by examining the deviation of split frequencies (approached zero), the overlay plot of generations versus log probability for multiple runs (which showed a clear plateau), the convergence diagnostics, and the potential scale reduction factors values (which were equal to 1). Group 1 is restricted to Central America. Group 2 is found in Venezuela, Trinidad and western Guyana. Groups 3 and 4 occupy almost sympatric distributions in Guyana and Suriname (Figure 3, see Table 1 for sequence divergences among groups). When all three genes are used, the maximum likelihood topology matches the Bayesian reconstruction recovered with all other methods (MrBayes [47], BEAST, *BEAST [48]) which is the topology used in all further analyses (Figure 4). 

**Table 1:** Mean pairwise COI sequence divergence estimated using the Kimura-2 parameter model of sequence divergence within and among 4 recognized groups

**Group**	**Group 1**	**Group 2**	**Group 3**	**Group 4**
Group 1	0.8%			
Group 2	5%,	0.5%		
Group 3	10%	11%	0.04%	
Group 4	11.2%	11.5%	5.1%	0.7%

### Congruence in mitochondrial, y-chromosome and RAG2 sequences

Among COI sequences we identified 124 variable positions yielding 47 unique haplotypes. In the *Dby* 7^th^ intron region we identified 13 variable positions and a 15 bp indel starting at the 214^th^ bp, which together yielded three unique haplotypes. In the RAG2 region we identified 12 variable positions yielding ten unique haplotypes.

**Figure 5:**
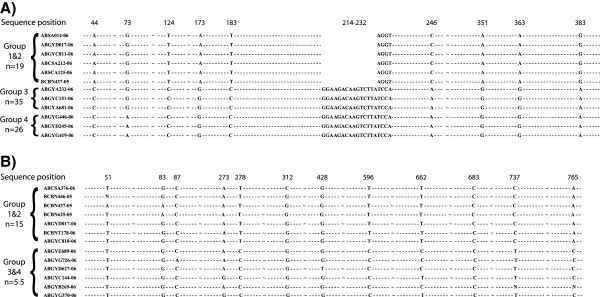
**Variable and fixed characters in A) the *Dby *7**^**th **^**intron and B) the RAG2 regions which differentiate mitochondrial lineages.** Base pair references are given above the sequences, ~ indicates removed sequence positions which contain no polymorphisms. Actual sample size for each group is indicated next to example sequences.

Three of the four groups identified by mtDNA also contained unique fixed characters in the *Dby* 7^th^ intron (n=80). The sequenced region was 443 bp long in Guyana/Suriname Groups 3 and 4. Group 3 (n=35) can be distinguished from Group 4 (n=26) by one fixed substitution at nucleotide position 73 (Figure 5). The allopatric Central American Group 1 (n=13) and Venezuela/Trinidad/Guyana Group 2 (n=6) show identical intron sequences, but contain a 15 nucleotide deletion and numerous polymorphisms relative to Groups 3 and 4 (Figure 5). A single specimen (ROM 114045) in this data set may represent a hybrid as we recovered the mitochondrial DNA of Group 3 and the y-chromosome sequence of Group 4 from this individual. We did not observe obvious double peaks in the electropherograms for RAG sequences, suggesting that heterozygosity is not a significant factor in our analysis. In the RAG2 region, Groups 1 and 2 can be distinguished from Groups 3 and 4 by fixed substitutions at nucleotide positions 278, 596, 737 and 765 (Figure 5).

### Estimates of divergence time

**Figure 6:**
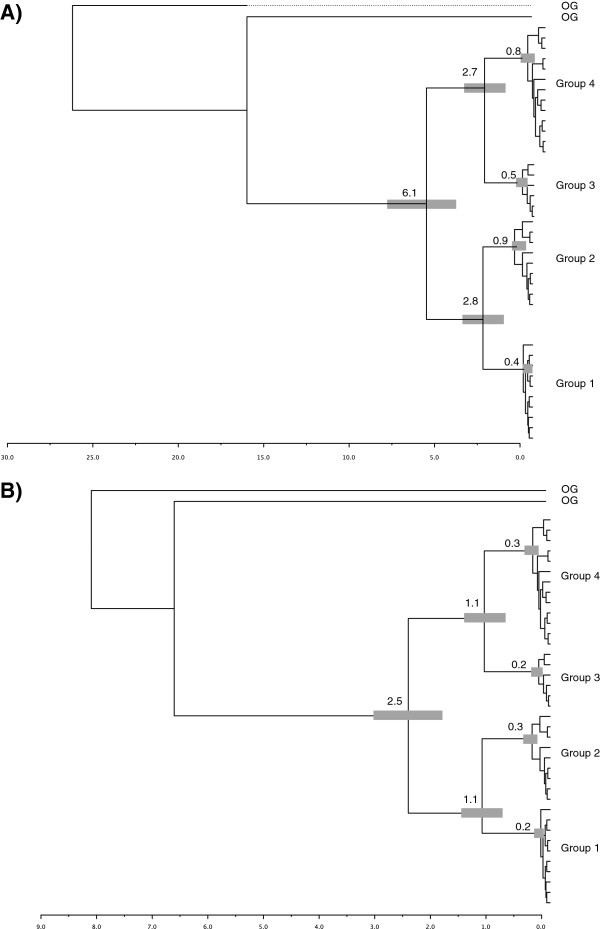
**Bayesian estimates of divergence time using two fixed substitution rates of 2% per million years (A) and 5% per million years (B) using phylogenetic reconstructions of unique COI haplotypes and the topology supported by the multi-gene reconstruction.** Estimated divergence dates (MYBP) are indicated, grey bars are scaled to represent 95% confidence intervals for estimated dates.

We estimated divergence times based on two fixed estimates of 2% (Figure 6A) and 5% (Figure 6B) divergence per million years in COI (Figure 6) and 0.194% in RAG2. Using the multi-gene topology (Figure 4), divergence dates for COI suggest Groups 3 and 4 diverged 1.1–2.7 million years before present (MYBP) while Groups 1 and 2 diverged 1.1 - 2.8 MYBP. The entire complex (Group (1+2) and Group (3+4)) last shared a common ancestor between 2.5 - 6.1 MYBP (Figure 6). Similar estimates are recovered from the RAG2 sequences with the divergence of Group 1+2 from 3+4 occurring approximately 5.4 MYBP (95% CI 3.5–7.8 MYBP) which is similar to the estimates of COI based on the higher mutation rate. We evaluated multiple runs in BEAST which converged on the same estimates.

### Analysis of morphology

No single morphological character analyzed can be used to distinguish these groups in the field (Table 2). Forearm differed significantly among groups (H_3_ = 74.30, p<0.001) with the exception of Group 2 which could not be differentiated from any other group. Forearm measurements overlap precluding the use of this character for field identification. Group 1: mean = 59.9±2.8 mm, range = 56–68 mm; Group 2: mean = 62.4±2.3, range = 59–65 mm; Group 3: mean = 63.2±1.4, range = 59–67 mm; Group 4: mean = 65.2±1.6, range = 61–69 mm.

**Table 2:** **Eleven cranial measures from *Pteronotus parnellii *used in this study**

	Condylobasal length*	**Zygomatic breadth**	**Breadth Of braincase**	**Mastoid breadth**	**Zygorostral length**	**Interorbital breadth**	**Rostral breadth**	**Alveolar length maxillary toothrow**	**Alveolar length mandibular toothrow**	**Breadth of post-palatal extension**	**Depth of braincase**
Group 1 N=27	20.69 (19.99–21.99)	12.35 (11.88–13.16)	10.51 (10.14–10.88)	11.45 (11.12–11.95)	15.71 (15.07–16.70)	4.35 (4.18–4.59)	8.00 (7.62–8.38)	9.15 (8.80–9.74)	9.76 (9.41–10.48)	1.60 (1.35–1.80)	8.84 (8.23–9.46)
Group 2 N=5	21.65 (21.47–21.92)	12.72 (12.25–12.93)	10.94 (10.77–11.18)	11.70 (11.44–12.04)	16.43 (16.30–16.51)	4.56 (4.42–4.80)	8.30 (8.16–8.48)	9.57 (9.48–9.62)	10.25 (10.16–10.44)	1.64 (1.51–1.71)	9.06 (8.79–9.28)
Group 3 N=60	21.38 (20.61–22.65)	12.81 (12.28–13.80)	10.77 (10.27–11.33)	12.06 (11.59–13.03)	16.16 (15.48–17.36)	4.61 (4.25–5.00)	8.56 (8.16–8.94)	9.44 (9.11–10.00)	10.03 (9.68–10.59)	1.67 (1.48–1.84)	9.13 (8.59–9.64)
Group 4 N=74	22.45 (20.84–23.23)	13.39 (12.52–14.01)	11.07 (10.61–11.42)	12.36 (11.54–12.91)	17.16 (15.79–17.70)	4.54 (4.17–4.92)	8.92 (8.09–9.35)	9.96 (9.22–10.32)	10.58 (9.86–10.99)	1.65 (1.43–1.84)	9.33 (8.78–9.69)

*includes incisors.

Means and ranges (mm) for each genetic grouping are given.

**Figure 7:**
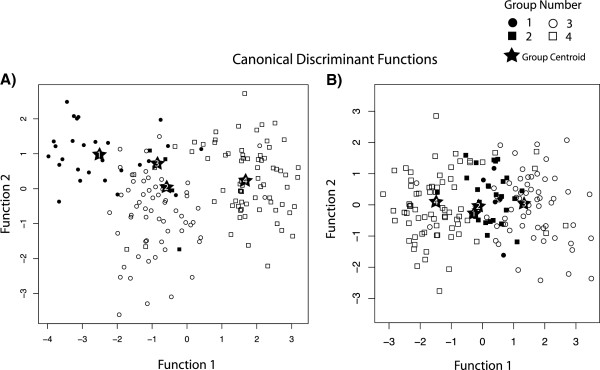
**Canonical discriminant function analysis (DFA) of genetic groups of *Pteronotus parnellii *shows significant divergence. (A)** Sympatric groups 3 and 4 show greater separation when the data are corrected for correlations with latitude. **(B)** Group centroids are marked with a star.

Using DFA we were able to distinguish the four groups based on two principal components (PC) scores which explained 87.2% of the variance. PC1 accounted for 76.9% of the variation and was highly weighted with all of the skull measurement variables of size such that low PC1 scores suggest smaller skulls. PC2 included shape components and explained 10.3% of the variation, such that a low PC2 score indicates short, wide skulls while a high PC2 score indicates long, narrow skulls. In the DFA, both PC1 and PC2 were significant (PC1: Eigenvalue = 2.575, Wilks λ = 0.199, p<0.001; PC2: Eigenvalue = 0.404, Wilks λ = 0.712, p<0.001). Leave-one-out cross-validation correctly classified 74.1% of Group 1, 80% of Group 2, 60% of Group 3, and 90.4% of Group 4 (Figure 7A). The DFA with the residual values from the regression of PC1 and PC2 against latitude was significant (Eigenvalue = 1.644, Wilks λ = 0.372, p<0.001). Leave-one-out-classification correctly classified 40% of Group 1, 44.4% of Group 2, 73.3% of Group 3, and 82.2% of Group 4, with Groups 1 and 2 being misclassified as each other 40% of the time (Figure 7B).

Females had generally smaller skulls (PC1: F_2,161_ = 10.12, p<0.001) that were also shorter and wider (PC2: F_2,159_ = 4.75, p=0.009). There were no interactions between sex and either latitude or group, which confirms that the pattern of sexual dimorphism was consistent among locations. For PC1 there was a significant interaction between group and latitude (F_3,155_ = 15.308, p<0.001). There were no significant interactions among sex, group and latitude in PC2. There was no effect of latitude on PC2 (F_1,159_ = 1.68, p = 0.197), but PC2 did vary significantly among groups (F_3,159_ = 24.66, p<0.001) where Group 3 differed from the other three groups.

### Divergence in acoustic recordings

There was a significant difference in the constant frequency of the second harmonic among three of the four groups (F_2,34_ = 125.403, p<0.001; Table 3a) and among the five sampled locations (F_4,39_ = 570.489, p<0.001, Table 3b). The constant frequency component of the calls was lowest in Guyana and highest in Central America, particularly in Belize. Among genetic groups, the DFA correctly classified 100% of the Guyana (Group 3/4) and Central American (Group 1) calls and 70.6% of the Trinidad (Group 2) calls (Eigenvalue = 7.377, Wilks λ = 0.119, p <0.001). The remaining 29.4% of Trinidad calls were misclassified as Central American in origin. Among locations, the DFA classified the calls of bats from all locations with 100% accuracy with the exception of calls recorded in Jamaica and Costa Rica, which did not differ significantly, causing misclassification between the two locations (Eigenvalue = 58.512, Wilks λ = 0.017, p <0.001).

**Table 3:** **Mean constant frequency (kHz±SD) for the second harmonic for*Pteronotus parnellii*in a) genetic groups and in b) the five sampled locations**

**Classification**	**N**	**Mean constant frequency (kHz)**
a) By Genetic Group		
Group 1	17	62.1±1.91b
Group 2	10	58.9±0.39a
Group 3 or 4*	10	53.6±0.30c
b) By Sample Location		
Belize	7	64.2±0.34 a
Costa Rica	10	60.5±0.32 b
Guyana	10	53.6±0.30 d
Jamaica (Type location)	7	60.6±0.92 b
Trinidad	10	58.9±0.39 c

Note: Means followed by the same letter are not significantly different according to Tukey’s HSD.

* Groups are sympatric thus call passes may belong to either (or both) groups.

## Discussion

In this study we have tested the hypothesis that *P. parnellii* consists of multiple undescribed species by exploring genetic, morphological, and acoustic divergences among four identified mitochondrial lineages. We demonstrate that intraspecific lineages identified with mitochondrial DNA are supported by fixed characters in a y-linked intron and the nuclear recombination activating gene 2 as well as by significant morphological differences. Acoustic variation is also evident within this complex, which corresponds to both genetic groups and geographical locations. Further, acoustic divergence is subtle, between 2.5 and 11 kHz, translating into wavelength differences of only 0.23–1.12 mm which are not likely to provide functional differences in resource use. This suggests that acoustic signals have diverged primarily through drift in allopatric populations or through selection for non-interference in sympatric groups rather than ecological selection for different prey sizes.

### A cryptic species complex

Mayer and von Helversen [23] showed that classification of bats based on morphological characteristics does not always correlate with mtDNA divergence. A similar pattern appears to be present here, where *P. parnellii* on the mainland harbours at least four genetically distinct taxa. These taxa appear to be morphologically cryptic without obvious characters which differentiate them in the field, though statistically significant morphological differences, acoustic variation, and genetic divergence support their existence. The groups do not appear to match the distributions reported for sub-species [31] (though see below). We have not included discrete character states in our morphological analysis (fur colour, banding pattern, etc.) as these are harder to quantify, but they may represent useful field characters for further investigation.

While any single uni-parentally inherited molecular marker has limitations (e.g. inability to assess hybridization), mtDNA can be a powerful tool for hypothesis generation in taxonomic research. In mammals, cytochrome *b* has historically been employed for similar analyses (e.g. [7,36,59]) though COI is favoured by DNA barcoding campaigns and has been employed extensively in Neotropical bats [8,9,25,26] allowing for easy comparison of sequence divergence (in particular see Clare *et al.*[8] for a review of COI sequence divergence in >9,000 individuals of 165 Neotropical bat species). Mitochondrial regions are frequently paired with one or more non-mitochondrial loci to test these hypotheses. Y-chromosome regions are ideal for comparison to mtDNA because they are fast evolving and non-recombining, but provide an exclusively paternal measure of gene flow. Particularly when male-biased gene flow is suspected (as is frequent in mammals; e.g. [60]) y-chromosome DNA can provide a rigorous test of the patterns observed in mtDNA (see [9]). Nuclear genes are also frequently used in phylogenetic analyses, but many are too slowly evolving for species level diagnosis, particularly when species are young. In addition, nuclear genes are bi-parentally inherited, raising problems of heterozygosity and recombination from multiple alleles. Nuclear genes can effectively support mitochondrial genes in phylogenetic reconstructions. In this case, the RAG2 region appears to provide limited but reliable support for a split between Groups (1+2) and (3+4) (Figure 5) and thus supports a phylogenetic topology which unites these groups (Figure 4). It should be noted that both the RAG2 and COI regions analyzed are protein-coding and thus subject to selection, however they have very different functions (immune system and electron transport chain respectively) resulting in different selection profiles. Additionally, neither region is known to be linked to any morphological trait, therefore they act as relatively independent markers.

In our analysis, all three genes provide evidence for a split between Groups (1+2) and Groups (3+4). The split between Groups 1 and 2 is not observed in the other two gene regions so we cannot evaluate the probability of male-biased gene flow but, as they are allopatric and acoustically distinct, the probability of hybridization is low. These two meet the criteria for the genetic species concept (GSC) advocated for mammals [7]. The GSC evaluates species based on the Bateson-Dobzhansky-Muller model and permits small amounts of gene flow if the genetic groups are on independent evolutionary trajectories [7]. Unlike the biological species concept, the GSC is applicable to allopatric populations and provides a framework for evaluating these groups. Finally, the y-chromosome supports the split between Groups 3 and 4. We were able to evaluate both mtDNA and the y-linked regions from these sympatric males and found little evidence of hybridization (n=1). This is lower than previously reported in other bat taxa, for example, Hoffman *et al.*[6] proposed cryptic species in *Uroderma bilobatum* and found two hybrids in 46 individuals and one potential F1, and in the European bats *Myotis myotis* and *M. blythii* introgression may be measured in 25% of individuals [61]. The low level of hybridization measured here is acceptable under the GSC and is also generally permitted under a relaxed biological species concept.

Though the *Dby* 7^th^ intron region has been amplified and sequenced in a wide variety of taxa [9,40], some caution is required in interpreting the data. While the region is fast evolving it does not appear to evolve as fast as mitochondrial DNA. Additionally, like mtDNA, it may be subject to reduced variability from selective sweeps. An homologous region has been identified on the X-chromosome in some species though it is substantially divergent [62]. We saw no evidence of co-amplification from the male X-chromosome and it is likely that it is either absent in *P. parnellii* or that these primers preferentially bind to the target area, though no rigorous testing has been done to our knowledge. The family Mormoopidae contains two genera: *Mormoops*, with three extant species, and *Pteronotus*, with seven extant species [63,64]. Of these, most are confined to Central America and the Antilles. *P. parnellii* appears to be the oldest lineage in the genus, and perhaps the family [38]. While all other species of *Pteronotus* may have a Central American origin [38] the origin of *P. parnellii* is still unclear though our phylogeny suggests a South American origin (see below).

We employed fixed clock estimates of 2% and 5% to exploit the upper and lower calibration points commonly used for bat divergence time calculations in mammal mtDNA. More precise calibrations have been used in other bat taxa, but these are taxonomically specific and estimated for cytochrome *b*. Given that COI is thought to evolve slightly slower than cytochrome *b*[65], using exact calibrations calculated for cytochrome *b* in bats would be inappropriate and we therefore employed minimum and maximum range bracketing. Previous calibrations have not been made for *P. parnellii* or COI in bats, thus our divergence rate calculations are meant to approximate the upper and lower estimates of divergence time in these analyses and to minimize errors in the divergence time estimates if any specific calibration point was used. The similar estimates from the RAG2 region suggest these are appropriate boundaries.

Using fixed clock estimates (Figure 4), Groups (1+2) and (3+4) diverged from a common ancestor 2.5- 6.1 MYBP during land bridge formation which ended ~3 MYBP [1] and a similar estimate was recovered from the RAG2 region. Groups 1, 2, 3 and 4 all radiated during or after the rise of the Eastern Cordillera 2–3 MYBP [2]. The most likely biogeographic scenario is that these groups have a South American origin as suggested by the phylogeny (a single invasion of Central America in the ancestor of Group 1) but invaded Central America during the great American interchange. The rise of the Eastern Cordillera effectively isolated the Central American population, giving rise to Group 1 in allopatry. However, our observations should be treated as preliminary. Additional samples from the Antilles region will be required to establish this definitively and to determine whether the invasion may have involved island hoping. Groups 2, 3 and 4 all exist within the Guyana Shield region which is one of the South American cratons [66,67]. While no obvious geological event correlates to the divergence, specimens in Group 2 are associated with higher elevations within the Guyana Highlands [68] though sampling here was minimal and the presence of the complex in Trinidad suggests more habitat variability. It is plausible that Group 2 has re-invaded South America either via the Antilles or a dispersal event over the Andes (additional sampling in Columbia, Venezuela, and the Antilles may resolve this). The divergence of Group 3 and 4 from a common ancestor occurred between 1.1 and 2.7 MYBP and both are associated with sympatric ranges at lower elevations within the Guyana shield region. While there is no contemporary pattern of geology or climatology that can provide a mechanism for this divergence, the region has experienced multiple climate shifts and continual forest change so divergence may have involved a past habitat restriction and vicariance event. Our sampling concentrates on the Central American and northern South American portions of the *P. parnellii* range and includes individuals from ten countries. We have not sampled individuals in the southern extent of their range, particularly Bolivia, Peru, and Central Brazil, or populations in the Caribbean which includes distributions of four additional subspecies [31]. Our intention was to sample areas encompassing all mainland subspecies however our results suggest that the distribution of these subspecies does not correspond with existing molecular divergences (see below). Additional sampling in these ranges will almost certainly uncover additional cryptic diversity and may help clarify the existence and ranges of these subspecies, their systematic status, and the origin and divergence of the complex.

### Systematic considerations in the complex

The taxonomic status of *P. parnellii* is exceedingly complex. Herd [31] recognized nine subspecies; *P. p. parnellii, P. p. gonavensis, P. p. portoricensis* and *P.p. pusillus* in the Antilles and *P. p. mexicanus, P. p. mesoamericanus, P. p. paraguanensis, P. p. fuscus* and *P.p. rubiginosus* on the mainland. Of these, *P. p. paraguanensis* was recently elevated to a species in Venezuela [64]. The most important systematic question regarding the groups recognized in this study is whether any may be considered *P. parnellii* sensu stricto. The type location for the taxon is Jamaica and is thought to encompass the subspecies *P. p. parnellii*. While we have been unable to include a genetic sample from this location in our analysis, the Jamaican population is acoustically distinct. We have some limited morphological data from Jamaica (not included), which demonstrates that this population is comprised of much smaller individuals than those on the mainland. For example, the forearm measures in Jamaica are 53 mm±1.26 SD (range 43–55, n=58, S. Koenig pers. comm.) which is non-overlapping with any mainland population we have examined. Additionally, a comparison of our cranial measurements with those presented for subspecies in Smith [53] indicate Group 1 best matches the measurements for *P. p. mesoamericanus* with little overlap among our measurements and those for *P. p. parnellii*. From this, we conclude that Group 1 corresponds with *P. p. mesoamericanus* and an elevation of that name would be appropriate. The individuals in our remaining groups are even larger thus none corresponds morphologically to *P. p*. *parnellii*. Suggesting appropriate names for the remaining three groups is more difficult as their distribution does not correspond with subspecies distributions suggested by Herd [31]. The remaining Antillean subspecies are even smaller than *P. p. parnellii* and their distributions disjunct, therefore these names are unlikely to be appropriate; likewise the distribution of *P. p. mexicanus* precludes a likely correspondence with Groups 2, 3 or 4. Of the remaining subspecies, *P. p. paraguanensis* (hereafter referred to by its species status)*, P. p. fuscus,* and *P. p. rubiginosus,* distributions from Herd [31] do not correspond with our analysis. In Venezuela, Gutierrez and Molinari [64] report that *P. paraguanensis* is significantly smaller than either *P. p. fuscus* or *P. p rubiginosus*, with *P. p. rubiginosus* the largest; however Gutierrez and Molinari [64] agreed with Herd [31] that only *P. p. rubiginosus* is found east of the Rio Orinoco suggesting that these subspecies do not correspond with any specific group identified here unless the distributions are much larger than previously reported. Thus, while we are confident that none of our genetic groups can rightly be called *P. parnellii*, we cannot, at this stage, suggest what names would be appropriate for Group 2, 3 and 4. In the interim, we suggest they be referred to as *Pteronotus* species 2, 3 and 4 until more appropriate binomials can be established. Barring contradictory evidence, we further conclude that *P. parnellii* be considered a taxon endemic to the Antilles. The most appropriate future analysis will be a molecular comparison between type material for these subspecies and the groups identified here with additional sampling in the Amazon and Antilles. See also Dávalos [69] for a discussion of diversification in this family.

### Divergence of morphological characters

Though forearm and cranium measurements are both correlated with overall size, we have treated them as independent in our statistical analysis as they have different applications. Cranial characters may only be measured accurately in extracted skulls (e.g. a museum collection), while forearm measurements can be easily obtained from live animals. As such, forearm length is frequently employed as a quick taxonomic field character. However, due to the high overlap in the forearm measurements of the groups, this would not be a reliable measure to separate these groups in the field. Our analysis indicates that the cranial characters advocated by Smith [53] for the family can differentiate our groups with reasonable accuracy and may act as a useful tool for further investigation of subspecies throughout the entire range, though some overlap does exist thus molecular evidence is a more reliable definitive character. While the four groups can be largely discriminated by DFA (87% successful), there is a significant effect of latitude on the main principal component (PC1-size). Once we corrected for the latitudinal cline, DFA continued to show the groups separating out based on cranial morphological characters. Differences between the two sympatric groups, 3 and 4, became more apparent, while divergence between Groups 1 and 2 was not as obvious after correction (Figure 7). This suggests that the morphological differences of Groups 3 and 4 to all other groups are not solely driven by the latitudinal cline, but this is less clear with Groups 1 and 2.

There was a strong effect of sex on both the size (PC1) and shape (PC2) of the skull suggesting some sexual dimorphism in all locations. The shape of the skull (PC2) did not show a latitudinal effect but did vary between Group 3 and all other groups, further suggesting that there are morphological differences among the groups that are not driven by latitude.

### Acoustic divergences within and between groups

Our analysis suggests that there is significant acoustic variation in these groups, creating distinct calls which can differentiate among Groups 1, 2 and 3/4. There is a steady decrease in the frequency of the call with decreasing latitude which also corresponds to an increase in the size of many morphological components.

Interestingly, enough variation is present to further distinguish between some regions within Group 1. We cannot conclude whether there are acoustic differences between the sympatric Groups 3 and 4, as we are limited by relatively few recordings from the region and an inability to separate free-flying bats in field recordings. Further sampling will be required in combination with molecular analysis to determine whether these two groups share an identical echolocation call or whether an additional acoustic pattern exists in this region. If it does, it could prove a powerful tool in identifying the two groups in the field and this is a clear goal for future field studies (sound files available from the authors on request). In addition to echolocation calls, it would be extremely valuable to examine the role of non-echolocation vocalizations among these groups. The role of “social calls” in intra- and interspecific recognition has not been well documented; however the degree of variation in social call repertoire is extensive and may play an important role in mate recognition.

The acoustic variation observed here is not consistent with the pattern of harmonic hopping observed by Kingston and Rossiter [29] in the *R. philippinensis* complex*,* but is similar to divergence patterns in the *H. bicolor* complex [33]. Individuals are unlikely to recognize the calls of other groups if they come into auditory contact. Furthermore, the divergence between calls is small and, as in *H. bicolor*, probably not sufficient to support ecological resource partitioning. As in *H. bicolor*, social character displacement and selection for non-interference may have played a large role in the diversification of the *P. parnellii* complex, particularly in northern South America where there are no obvious barriers to prevent contact between all three groups. If the Central American group has diverged in allopatry, it is likely that the variation in the constant frequency component has arisen by drift. It is not known what threshold of variation is used for social recognition in *P. parnellii,* but estimates from Old World bats suggest that the constraints of high-duty cycle echolocation limit the possible variation more than in low-duty cycle species [33]. Future research on inter- and intraspecific call divergence and the social aspects of call recognition and discrimination will prove interesting and may support an allopatric or sympatric origin of these groups.

### Echolocation: a mode of reinforcement and speciation?

Theoretically, for speciation to occur despite substantial gene flow, reinforcement and a strong mate choice character are almost certainly required. In bats, non-morphological traits may be very important in speciation. In some potential cryptic species complexes, for instance *Saccopteryx bilineata* (family Emballonuridae) [9], olfactory cues may be particularly important. In bats that rely on echolocation, divergence between echolocation calls may reduce signal competition and influence prey selection [29,70] but also change mate recognition [71], creating an almost instantaneous form of pre-zygotic isolation. For reinforcement to directly influence speciation, a genetic association (linkage) between the ecological trait under selection and the mating signal is required [70,72], a situation which should be disrupted by recombination [73]. This problem is averted if the diverging trait is controlled by the same genetic loci which become fixed in the diverging populations (one allele model of Felsenstein [73]). This is particularly effective when the characters governing both pre- and post-zygotic isolation are the same, so-called “magic traits” [74,75], and direct selection has pleiotropic effects [72]. Echolocation may meet both of these criteria [75] and, in this context, changes in echolocation could rapidly lead to speciation.

Ecological character displacement (selection for increased ecological niche separation which indirectly influences mate choice [72]) may also speed the process of differentiation. The divergence of echolocation call design may start as selection against signal interference between populations [29,33] but may be taken over by selection for mate recognition. In this scenario, speciation can be swift in the presence of gene flow whether selection is initially acting on niche specialization or mate recognition [70,76].

In our analysis, Groups 3 and 4 currently occupy sympatric distributions (Figure 3). Elmer and Meyer [77] outline four “gold standard” criteria for the hypothesis of sympatric speciation: 1) sympatric distributions, 2) a reciprocally monophyletic relationship between the taxa (sister species) with respect to others in the complex, 3) reproductive isolation and, 4) a setting where allopatric divergence is unlikely. Groups 3 and 4 appear to meet at least the first three criteria, but several lines of evidence could refute sympatric speciation. First, the relationship between individuals from the Brazilian Amazon and French Guiana is unknown. If a group from these areas is sister to Group 3 or Group 4 (raising the possibility of historical allopatric ranges), the hypothesis of sympatric speciation may be refuted. Additionally, pre-zygotic isolation should develop before post-zygotic isolation in cases of sympatric speciation [78]. The presence of only one hybrid suggests that hybrids are rare but does not provide evidence for the cause. Searching for evidence of F2 individuals could illuminate this [78].

## Conclusions

The present study has established that *P. parnellii* from the mainland regions of Central and northern South America represents four distinct species with distinct genetic characters and divergent morphology and echolocation. With the exception of a single potential hybrid, they appear to be reproductively isolated and meet the biological species concept [79] and certainly meet the genetic species concept as outlined for mammals [7]. We suggest the elevation of *P. mesoamericanus* as an appropriate name for individuals in Central America, however the taxonomy in South America remains highly complex and none of these species correspond with *P. parnellii*. High-duty cycle echolocation has always been considered an Old World trait among bats with only this single example in the New World. The presence of at least five high-duty cycle species with a single origin in the New World presents an interesting case of adaptive radiation in only a few million years. This complex is also an excellent, convergently-evolved model for comparison with species undergoing rapid radiation via high-duty cycle echolocation in the Old World. The potential of this system for the study of allopatric and sympatric divergence makes it a very exciting complex for both evolutionary and ecological research.

## Competing interests

The authors declare that there are no competing interests.

## Authors’ contributions

ELC and AMA conceived of the project, conducted field work, performed analysis and co-wrote the manuscript. AZMS performed morphological examinations. JLE provided all specimens and tissues from the ROM and supervised morphological examinations. PDNH and MBF assisted with the design of the projects and the writing of the manuscript and provided funding for the project. All authors read and approved the final manuscript.

## Supplementary Material

Additional file 1 Specimen accession numbers for BOLD and Genbank. Click here for file

Additional file 2 COI alignment. Click here for file

Additional file 3 DBY alignment. Click here for file

Additional file 4 RAG2 alignment. Click here for file

Additional file 5 **Primer Sequences [**[80]**-**[84]**].** Click here for file
