# Supplementary material for: Diversification and reproductive isolation: cryptic species in the only New World high-duty cycle bat, Pteronotus parnellii
Source: BMC Evol Biol. 2013 Jan 29;13:26. doi: 10.1186/1471-2148-13-26 (PMC3567945; doi:10.1186/1471-2148-13-26)
Supplement: Additional file 5 — Primer Sequences [[80]-[84]]. [file 1471-2148-13-26-S5.doc]

Supplemental File 5: Primer sequences

| **PCR Cocktail** | **Primer Sequences** | **Reference** |
| --- | --- | --- |
| **COI Primers** |  |  |
| C_VF1di + C_VR1di (F) | VF1 5’-TTCTCAACCAACCACAAAGACATTGG-3’ | 80 |
| (Cocktail) | VF1d 5’-TTCTCAACCAACCACAARGAYATYGG-3’ | 80 |
|  | VF1i 5’-TTCTCAACCAACCAIAAIGAIATIGG-3’ | 80 |
| C_VF1di + C_VR1di (R) | VR1 5’-TAGACTTCTGGGTGGCCAAAGAATCA-3’ | 80 |
| (Cocktail) | VR1d 5’-TAGACTTCTGGGTGGCCRAARAAYCA-3’ | 80 |
|  | VR1i 5’-TAGACTTCTGGGTGICCIAAIAAICA-3’ | 80 |
| C_VF1LFt1 + C_VR1LRt1 (F) | LepF1_t1 5’-TGTAAAACGACGGCCAGTATTCAACCAATCATAAAGATATTGG-3’ | 80 |
| (Cocktail) | VF1_t1 5’-TGTAAAACGACGGCCAGTTCTCAACCAACCACAAAGACATTGG-3’ | 81 |
|  | VF1d_t1 5’-TGTAAAACGACGGCCAGTTCTCAACCAACCACAARGAYATYGG-3’ | 81 |
|  | VF1i_t1 5’-TGTAAAACGACGGCCAGTTCTCAACCAACCAIAAIGAIATIGG-3’ | 81 |
| C_VF1LFt1 + C_VR1LRt1 (R) | LepR1_t1 5’- CAGGAAACAGCTATGACTAAACTTCTGGATGTCCAAAAAATCA-3’ | 81 |
| (Cocktail) | VR1_t1 5’- CAGGAAACAGCTATGACTAGACTTCTGGGTGGCCAAAGAATCA-3’ | 81 |
|  | VR1d_t1 5’- CAGGAAACAGCTATGACTAGACTTCTGGGTGGCCRAARAAYCA-3’ | 81 |
|  | VR1i_t1 5’- CAGGAAACAGCTATGACTAGACTTCTGGGTGICCIAAIAAICA-3’ | 81 |
| RonM | RonM 5’- GGMGCMCCMGATATRGCATTCCC-3’ | 82 |
| RonM_t1 | RonM 5’- TGTAAAACGACGGCCAGTGGMGCMCCMGATATRGCATTCCC-3’ | 25 |
| Sequencing-C_VF1di + C_VR1di (F) | VF1d 5’-TTCTCAACCAACCACAARGAYATYGG-3’ | 80 |
| Sequencing-C_VF1di + C_VR1di (R) | VR1d 5’-TAGACTTCTGGGTGGCCRAARAAYCA-3’ | 80 |
| Sequencing-C_VF1LFt1+C_VR1LRt1 (F) | M13F 5’- TGTAAAACGACGGCCAGT-3’ | 83 |
| Sequencing-C_VF1LFt1+C_VR1LRt1 (R) | M13R 5’- CAGGAAACAGCTATGAC-3’ | 83 |
|  |  |  |
| **Dby 7^th^ intron region primers** |  |  |
| HDBY7F | HDBY7F 5’- GGTCCAGGAGARGCTTTGAA-3’ | 84 |
| HDBYR | HDBY7R 5’- CAGCCAATTCTCTTGTTGGG-3’ | 84 |
|  |  |  |
| **RAG2 Primers** |  |  |
| F1B | F1B 5’- ATCCTGCCCCACTGGAGTTTTC-3’ | 41 |
| R1 | R1 5’ - AACYTGYTTATTGTCTCCTGGTATGC-3’ | 41 |
